# Angiotensin II Induces Oxidative Stress and Endothelial Dysfunction in Mouse Ophthalmic Arteries via Involvement of AT1 Receptors and NOX2

**DOI:** 10.3390/antiox10081238

**Published:** 2021-08-02

**Authors:** Michael Birk, Ewa Baum, Jenia Kouchek Zadeh, Caroline Manicam, Norbert Pfeiffer, Andreas Patzak, Johanna Helmstädter, Sebastian Steven, Marin Kuntic, Andreas Daiber, Adrian Gericke

**Affiliations:** 1 Department of Ophthalmology, University Medical Center, Johannes Gutenberg University Mainz, Langenbeckstrasse 1, 55131 Mainz, Germany; michael.birk@med.uni-tuebingen.de (M.B.); ebaum@ump.edu.pl (E.B.); je.kouchek@hotmail.de (J.K.Z.); caroline.manicam@unimedizin-mainz.de (C.M.); norbert.pfeiffer@unimedizin-mainz.de (N.P.); 2 Department of Ophthalmology, University Eye Hospital Tübingen, Elfriede-Aulhorn-Straße 7, 72076 Tübingen, Germany; 3 Department of Social Sciences and the Humanities, Poznan University of Medical Sciences, ul. Rokietnicka 7, 60-806 Poznań, Poland; 4 Institute of Vegetative Physiology, Charité-Universitätsmedizin Berlin, Charitéplatz 1, 10117 Berlin, Germany; andreas.patzak@charite.de; 5 Department of Cardiology, Cardiology I-Laboratory of Molecular Cardiology, University Medical Center, Johannes Gutenberg University, Building 605, Langenbeckstr. 1, 55131 Mainz, Germany; johanna.helmstaedter@uni-mainz.de (J.H.); sesteven@uni-mainz.de (S.S.); mkuntic@students.uni-mainz.de (M.K.); daiber@uni-mainz.de (A.D.)

**Keywords:** angiotensin II, endothelial dysfunction, ophthalmic artery, oxidative stress

## Abstract

Angiotensin II (Ang II) has been implicated in the pathophysiology of various age-dependent ocular diseases. The purpose of this study was to test the hypothesis that Ang II induces endothelial dysfunction in mouse ophthalmic arteries and to identify the underlying mechanisms. Ophthalmic arteries were exposed to Ang II in vivo and in vitro to determine vascular function by video microscopy. Moreover, the formation of reactive oxygen species (ROS) was quantified and the expression of prooxidant redox genes and proteins was determined. The endothelium-dependent artery responses were blunted after both in vivo and in vitro exposure to Ang II. The Ang II type 1 receptor (AT1R) blocker, candesartan, and the ROS scavenger, Tiron, prevented Ang II-induced endothelial dysfunction. ROS levels and NOX2 expression were increased following Ang II incubation. Remarkably, Ang II failed to induce endothelial dysfunction in ophthalmic arteries from NOX2-deficient mice. Following Ang II incubation, endothelium-dependent vasodilation was mainly mediated by cytochrome P450 oxygenase (CYP450) metabolites, while the contribution of nitric oxide synthase (NOS) and 12/15-lipoxygenase (12/15-LOX) pathways became negligible. These findings provide evidence that Ang II induces endothelial dysfunction in mouse ophthalmic arteries via AT1R activation and NOX2-dependent ROS formation. From a clinical point of view, the blockade of AT1R signaling and/or NOX2 may be helpful to retain or restore endothelial function in ocular blood vessels in certain ocular diseases.

## 1. Introduction

The renin-angiotensin system (RAS) plays a critical role in blood pressure regulation [1]. Elevated plasma levels of angiotensin II (Ang II) were reported to increase arterial pressure, produce oxidative stress and impair endothelial function [2,3,4,5]. It was shown in mice that Ang II induces a pro-inflammatory phenotype that is characterized by the infiltration of monocytes into the vascular wall as well as higher levels of reactive oxygen species (ROS) [6]. Of note, the genetic ablation of lysozyme M-positive myelomonocytic cells prevented Ang II-mediated hypertension, endothelial dysfunction, and vascular inflammation, as well as oxidative stress. Whereas adoptive cell transfer of wildtype monocytes to the ablated mice restored all adverse effects of Ang II, the transfer of monocytes from mice lacking the Ang II type 1 receptor (AT1R) or the phagocytic NADPH oxidase (NOX2) failed to restore hypertension and other complications, supporting a central role of Ang II type 1 receptor (AT1R)-NOX2 signaling for Ang II-conferred adverse effects [6]. Since it was shown that endothelial and vascular dysfunction both progress with age as well as with the burden of oxidative stress, e.g., by genetic deletion of antioxidant enzymes, such as manganese superoxide dismutase or glutathione peroxidase-1, the aging process and accumulation of oxidative damage may also impair ophthalmic endothelial and vascular function [7,8]. As the aging process, per se, is associated with higher ROS formation rates, especially from mitochondria, these “oxidative stress aging conditions” can be mimicked by a deficiency in antioxidant enzymes, e.g., manganese superoxide dismutase, leading to more pronounced eNOS uncoupling, endothelial dysfunction, and higher blood pressure in Ang II-treated manganese superoxide dismutase deficient mice [9].

Receptors for Ang II have also been identified on blood vessels supplying the retina and optic nerve [10,11,12]. Moreover, responses to Ang II have been elicited in ocular blood vessels of various species in vitro and in vivo [13,14,15,16]. Apart from its effects on the vascular tone, Ang II was shown to mediate various other pathophysiological actions in ocular blood vessels, such as proliferation and migration of smooth muscle cells and pericytes, uptake of glucose into retinal pericytes, increase of vascular endothelial growth factor (VEGF) expression, and potentiation of VEGF-induced angiogenic activity [17,18,19,20,21,22]. Blockade of the RAS was shown to stop or delay the breakdown of the blood–retina barrier and to prevent retinal neovascularization in diabetic retinopathy and in retinopathy of prematurity [23,24,25,26,27]. Favorable effects of RAS blockade have also been reported for age-related macular degeneration, autoimmune uveitis, ischemia-reperfusion (I/R) injury, and glaucoma [28,29,30,31,32,33,34]. Although numerous studies support the involvement of Ang II in the pathophysiology of certain ocular diseases, little is known of the effects of Ang II on endothelial function in ocular blood vessels. Hence, the rationale for the present study was to test the hypothesis that Ang II impairs endothelium-dependent vasodilation in ocular blood vessels. Another objective of this study was to investigate the potential mechanisms of action. For that reason, we established an in vitro model for studying the effects of Ang II in isolated ophthalmic arteries, which is based on a previously reported protocol for larger blood vessels [35,36].

## 2. Materials and Methods

### 2.1. Animals

All animals were treated in accordance with the guidelines of the EU Directive 2010/63/EU for animal experiments and were approved by the Animal Care Committee of Rhineland-Palatinate, Germany (approval number: 23 177-07/G 17-1-050 and 23 177-07/G 16-1-055 with extensions E1-E3). All experiments were performed in male, 2-4-month-old C57BL/6J mice. In one series of experiments, Ang II (0.5 mg/kg/d for 7 days, Bachem Holding AG, Bubendorf, Switzerland) versus sham (NaCl 0.9%) was administered subcutaneously via mini-osmotic pumps (model 1007D, ALZET, DURECT Corporation, Cupertino, CA, USA), and implanted under ketamine/xylazine anesthesia as described previously [37]. One week after pump implantation, animals were sacrificed by cardiac puncture after sedation with isoflurane and anesthesia with ketamine/xylazine (80 mg/kg ketamine and 12 mg/kg xylazine in 0.9% NaCl) for organ isolation. The ophthalmic arteries used in the present study were harvested from mice included in the study by Helmstadter et al. [37]. The mice developed arterial hypertension and endothelial dysfunction in their aortic rings, indicative of systemic Ang II-induced effects [37].

To test whether the in vivo effects of Ang II on ophthalmic artery reactivity can be mimicked in vitro, isolated ophthalmic arteries were exposed to different concentrations of Ang II or vehicle in cell culture medium. For some experiments, mice deficient in the gene coding for NOX2 (gp91phox) (NOX2^–/–^) of the C57BL/6J background (Jackson Laboratories, Bar Harbor, ME) were used [38]. Mice were housed under pathogen-free conditions with a 12 h light/dark cycle, a temperature of 22 ± 2 °C, a humidity of 55 ± 10%, and with ad libitum standard rodent chow (Altromin, Lage, Germany) and tap water.

### 2.2. Preparation and Incubation of Ophthalmic Arteries

After the mice had been killed, the eyes with the retrobulbar tissue were excised and placed in cold Krebs Henseleit buffer. Next, ophthalmic arteries were isolated under a dissecting microscope, cleaned from the surrounding tissue, and subjected for measurement of vascular reactivity by video microscopy as described previously [39]. For in vitro incubation experiments, the isolated ophthalmic artery tree was cannulated from the proximal end with a micropipette and perfused with Dulbecco’s Modified Eagle Medium (DMEM, Merck, Darmstadt, Germany) containing 5.5 mmol/l glucose, 120 U/mL penicillin, 120 µg/mL streptomycin, and either Ang II (10^−9^ M, 10^−8^ M or 10^−7^ M, Sigma-Aldrich, Taufkirchen, Germany) or vehicle (NaCl 0.9%). Transluminal perfusion was conducted to remove blood cells from the lumen of vessels and to ensure direct exposure of endothelial cells to angiotensin II or vehicle solution. After 30 s of perfusion, the ophthalmic artery tree was removed from the micropipette and placed in a NunclonTM cell culture dish of 35 mm diameter (Thermo Fisher Scientific, Dreieich, Germany) containing 2.0 mL of DMEM of the same composition that had been used to perfuse the vessels. Arteries were then incubated for 22 h at 37 °C (5% CO_2_) as previously described for larger blood vessels [35,36].

### 2.3. Measurement of Vascular Reactivity

For functional studies, the ophthalmic arteries were transferred to an organ chamber filled with cold Krebs buffer, then cannulated and sutured onto two micropipettes, as described previously with minor modifications [39]. Vessels were pressurized via the micropipettes to 50 mmHg under no-flow conditions by using two reservoirs filled with Krebs buffer and visualized using a video camera mounted on an inverted microscope. Video sequences were captured by a personal computer for offline analysis. The organ chamber was continuously circulated with oxygenated and carbonated Krebs buffer at 37 °C and pH 7.4. Arteries were equilibrated for 30 min before the study. The viability of vessels was regarded as satisfactory when at least 50% constriction from resting diameter in response to high KCl solution (100 mM) was achieved. The aim of the first series of experiments was to determine whether Ang II induces endothelial dysfunction in ophthalmic arteries when applied in vivo. For that reason, ophthalmic arteries were harvested from mice that had received either Ang II (0.5 mg/kg/d, *n* = 8) or NaCl (0.9%, *n* = 8) subcutaneously for 7 days. After mice had been sacrificed and the vessels isolated, cumulative concentration-response curves for the thromboxane mimetic, 9,11-dideoxy-9α,11α-methanoepoxy prostaglandin F2α (U46619, 10^−11^ M to 10^−6^ M; Cayman Chemical, Ann Arbor, MI, USA) were performed. For testing vasodilatory responses, vessels were preconstricted to 70–50% of the initial luminal diameter by titration of U46619. After preconstriction, responses to the endothelium-independent nitric oxide (NO) donor, sodium nitroprusside (SNP, 10^−9^ M to 10^−4^ M, Sigma-Aldrich) and to the endothelium-dependent vasodilator, acetylcholine (10^−9^ M to 10^−4^ M; Sigma-Aldrich) were measured. We previously demonstrated in ophthalmic arteries from mice of different genetic backgrounds, including C57BL/6J mice, that acetylcholine induces only significant vasodilation when the endothelium is intact [40,41,42]. Between each concentration–response curve, the perfusion system was washed with Krebs buffer.

In a second series of experiments, responses to U46619, acetylcholine, and to SNP were obtained in arteries that had been preincubated with different concentrations of Ang II (10^−9^ M, 10^−8^ M, or 10^−7^ M) or vehicle (*n* = 8 per group) for 22 h to determine whether Ang II may induce endothelial dysfunction in ophthalmic arteries in vitro.

In a third series of experiments, ophthalmic arteries were preincubated for 22 h with Ang II (*n* = 8), with Ang II and the AT1R blocker, candesartan (10^−5^ M, Tocris Bioscience, Bristol, UK, *n* = 8), with vehicle (*n* = 8), or with vehicle and candesartan (*n* = 8), to determine whether the effects of Ang II on ophthalmic artery reactivity are mediated by the AT1R. Also, the role of the AT2R has been tested by preincubation for 22 h with Ang II (*n* = 8), Ang II and the AT2R blocker, PD 123319 ditrifluoroacetate (10^−6^ M, Tocris Bioscience, *n* = 8), vehicle (*n* = 8), or vehicle with PD 123319 ditrifluoroacetate (*n* = 8).

A fourth series of experiments was conducted to determine the involvement of reactive oxygen species (ROS) in the effects of Ang II on vascular reactivity. For that reason, ophthalmic arteries were preincubated for 22 h with Ang II (*n* = 8), Ang II and the ROS scavenger, Tiron (10^−3^ M, Sigma-Aldrich, n = 8), vehicle (*n* = 8), or with vehicle and Tiron (*n* = 8). To identify the potential source of ROS, functional studies were performed in ophthalmic arteries from NOX2^–/–^ mice that had been preincubated with either Ang II (*n* = 8) or vehicle (*n* = 8).

A fifth series of experiments was performed to test whether Ang II exposure affected endothelial vasodilatory signaling pathways. To assess the role of nitric oxide synthase (NOS) and cyclooxygenase (COX) metabolites in mediating endothelium-dependent ophthalmic artery vasodilation, the responses of arteries preincubated with Ang II or vehicle for 22 h were tested to acetylcholine (10^−9^ M to 10^−4^ M) before and after incubation (30 min) with the non-isoform selective COX inhibitor, indomethacin (10^−5^ M, Sigma-Aldrich, *n* = 8 per group), the non-isoform selective NOS inhibitor, Nω-nitro-L-arginine methyl ester (L-NAME, 10^−4^ M, Sigma-Aldrich, *n* = 8 per group), or a combination of both agents (*n* = 8 per group). To investigate the contribution of endothelium-derived hyperpolarizing factors (EDHFs) to endothelium-dependent vasodilation, ophthalmic arteries preincubated with Ang II or vehicle for 22 h were incubated with indomethacin (10^−5^ M) and L-NAME (10^−4^ M) without or with the addition of 30 mM of potassium (K+) solution to prevent hyperpolarization (*n* = 8 per group). To identify the specific EDHF signaling pathways, ophthalmic arteries that had been preincubated with either Ang II or vehicle for 22 h were incubated with the suicide substrate inhibitor of both ω-hydroxylation and epoxygenation of arachidonic acid via the cytochrome P450 oxygenase (CYP450) pathway, 17-octadecynoic acid (17-ODYA, 10^−4^ M, Tocris Bioscience, *n* = 8 per group), or the 12/15-lipoxygenase (12/15-LOX) inhibitor, baicalein (10^−5^ M, Sigma-Aldrich, *n* = 8 per group). In addition to the inhibitors both L-NAME (10^−4^ M) and indomethacin (10^−5^ M) were present in the organ bath to prevent the formation of NOS and COX metabolites, respectively. Each inhibitor was incubated for 30 min.

### 2.4. Quantification of Reactive Oxygen Species

ROS formation was determined in cryosections of the ophthalmic artery (one vascular cross-section per mouse) by dihydroethidium (DHE, 1 µM)-derived fluorescence. After incubation for 22 h in DMEM with Ang II or vehicle, ophthalmic arteries were washed for 30 s in phosphate-buffered saline (PBS), fixed in Tissue-Tek O.C.T. Compound (Sakura Finetek Europe, Alphen aanden Rijn, Netherlands), and frozen in liquid nitrogen. Freshly prepared cryosections (10 µm thickness) were incubated with 1 μM of dihydroethidium (DHE, Thermo Fischer Scientific, Waltham, MA, USA) for 30 min at 37 °C. By using an Eclipse TS 100 microscope (Nikon, Yurakucho, Tokyo, Japan) equipped with a DS–Fi1-U2 digital microscope camera (Nikon) and the imaging software NIS Elements (Nikon, Version 1.10 64 bit) the fluorescence (518 nm/605 nm excitation/emission) was recorded and measured in the vascular wall by using ImageJ (NIH, http://rsb.info.nih.gov/ij/) accessed on 11 March 2019 [43,44].

### 2.5. Quantification of Prooxidant Redox Genes by Real-Time PCR

To test, whether incubation of ophthalmic arteries with Ang II influenced the expression of prooxidant redox genes, mRNA for the prooxidant redox enzymes, nicotinamide adenine dinucleotide phosphate oxidase,1, 2, and 4 (NOX1, NOX2, NOX4), and for xanthine dehydrogenase (XDH) was quantified using quantitative real-time PCR (qPCR). Under physiological conditions, XDH is active primarily as a dehydrogenase. However, various stimuli, including hypoxia and inflammation, promote the conversion of XDH to the superoxide-producing xanthine oxidase (XO), which promotes endothelial dysfunction and atherosclerosis [45].

After incubation for 22 h with Ang II or vehicle, ophthalmic arteries were washed in cold PBS, transferred into a 1.5 mL tube, immediately snap-frozen in liquid nitrogen, and stored at −80 °C for 2–3 months. Next, the tissue was homogenized in lysis buffer (1.0% NP40, 0.5% sodium deoxycholate, 0.1% SDS, 10 mmol/l NaF, 80 mmol/l TRIS, pH 7.5). By using a light cycler (LC480, Roche Diagnostics, Mannheim, Germany) and a StepOnePlus device (Applied Biosystems, Foster City, CA, United States) qPCR was conducted according to the manufacturer’s protocol. We used the following cycling conditions: holding stage (initial denaturation): 10 min at 95 °C; cycling stage (×40): 20 sec at 95 °C and 40 sec at 60 °C; melt curve stage: 60 °C increased by 0.5 °C every 5 sec to 95 °C.

SYBR Green was used for fluorescent detection of generated DNA during qPCR. Relative mRNA levels of target genes were quantified using comparative threshold (CT) normalized to the β-actin gene (*ACTB*). Messenger RNA expression is presented as the fold-change in Ang II-incubated vessels versus vehicle-incubated vessels. Primers for mouse *ACTB*, *NOX1*, *NOX2*, *NOX4* and *XDH* were used. The qPCR primers were synthesized by TIB Molbiol, Berlin, Germany. Primer accession numbers and sequences are listed in Table 1.

### 2.6. Immunofluorescence Analysis

An antibody directed against NOX2 (ab129068, rabbit monoclonal, Abcam, Waltham, MA, USA, dilution 1:200) was applied to ophthalmic artery cryosections of 10 µm thickness to quantify NOX2 expression in the vascular wall. Tissue sections were fixed for 20 min in paraformaldehyde (4%) and incubated with bovine serum albumin (1%) for 30 min followed by the primary antibody for 2 h at room temperature. After washing the slides in PBS (3 × 5 min), a Rhodamine Red-X-coupled secondary antibody (111-295-003, goat anti-rabbit, polyclonal, Dianova GmbH, Hamburg, Germany, dilution 1:200) was applied for 1 h at room temperature. Tissue from NOX2^–/–^ mice served as the negative control. Finally, slides were washed in PBS (3 × 5 min) and were mounted using VECTASHIELD^®^ Mounting Medium with DAPI (BIOZOL Diagnostica Vertrieb GmbH, Eching, Germany) and cover-slipped. Subsequently, the fluorescence was measured in the vascular wall by using Image J (NIH, http://rsb.info.nih.gov/ij/ (accessed on 11 March 2019) as reported previously [44,46].

### 2.7. Statistical Analysis

Data are presented as mean ± SE, and n represents the number of mice per group. Constriction responses to U46619 are presented as percent change in luminal diameter from resting diameter, while responses to acetylcholine and SNP are presented as percent change in luminal diameter from the preconstricted diameter. Comparison between concentration-responses was made using two-way ANOVA for repeated measurements. For comparisons of multiple groups, Tukey’s multiple comparisons test was used. For comparisons of mRNA expression levels and fluorescent intensity, an unpaired t-test was used. The level of significance was set at 0.05.

## 3. Results

### 3.1. Effects of Ang II on Endothelial Function

The initial luminal diameter of ophthalmic arteries from mice subjected to Ang II (0.5 mg/kg daily for 7 days) did not differ from the diameter of arteries from mice subjected to NaCl 0.9% (96.13 ± 7.558 µm versus 102.0 µm ± 7.865 µm, *n* = 8 per group, *p* = 0.5986, unpaired t-test). After an equilibration time of 30 min, the luminal diameter was reduced by 11.48 ± 2.436% and by 14.58 ± 3.338% in ophthalmic arteries from Ang II-exposed and vehicle-exposed mice, respectively (*n* = 8 per group, *p* = 0.4663, unpaired t-test).

**Figure 1:**
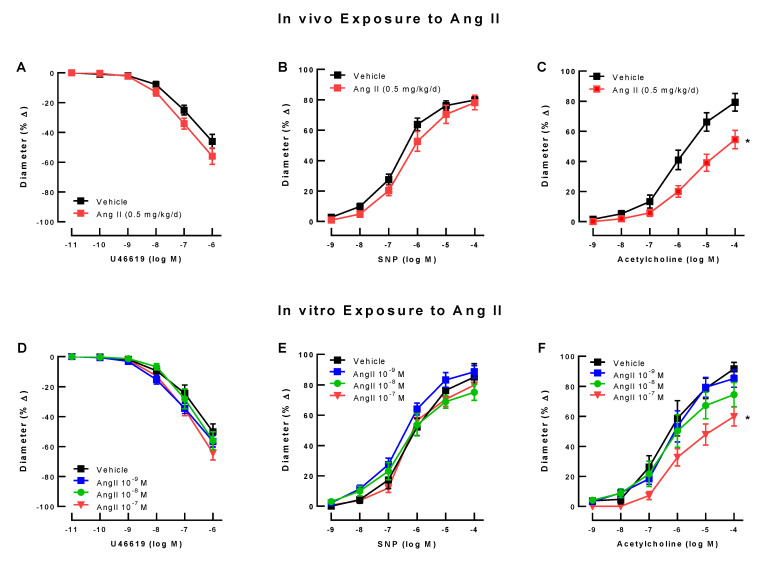
In vivo exposure to Ang II (0.5 mg/kg daily for 7 days) did not affect the response of ophthalmic arteries to the vasoconstrictor, U46619 (**A**), or to the endothelium-independent vasodilator, SNP (**B**), whereas it did cause a substantial degree of endothelial dysfunction (impaired acetylcholine response, (**C**)). Values are expressed as mean ± SE (*n* = 8 per group; * *p* < 0.05, Ang II 0.5 mg/kg/d versus vehicle control). In vitro exposure of ophthalmic arteries to different concentrations of Ang II also had no effect on the response to U46619 (**D**) or SNP (**E**), but markedly blunted reactivity to acetylcholine at 10^−7^ M of Ang II (**F**), indicative of endothelial dysfunction. Values are expressed as mean ± SE (*n* = 8 per group; * *p* < 0.05, Ang 10^−7^ M versus vehicle control).

U46619 (10^−11^ M–10^−6^ M) elicited concentration-dependent vasoconstriction that did not differ between ophthalmic arteries from mice subjected to Ang II (0.5 mg/kg daily for 7 days) and sham controls subjected to NaCl 0.9% (Figure 1A). There were also no differences between both mouse groups in response to SNP (Figure 1B). In contrast, responses to the endothelium-dependent vasodilator, acetylcholine, were markedly reduced in mice treated with Ang II, indicative of endothelial dysfunction (Figure 1C). The maximum increase in luminal diameter to acetylcholine was 79.28 ± 5.857% and 54.53 ± 6.096% in arteries from Ang II-infused and vehicle-infused mice, respectively.

In ophthalmic arteries preincubated with Ang II 10^−9^ M, Ang II 10^−8^ M, Ang II 10^−7^ M or vehicle, respectively, vasoconstrictor responses to U46619 (10^−11^ M to 10^−6^ M) were similar (Figure 1D). Responses to the endothelium-independent vasodilator, SNP (10^−9^ M to 10^−4^ M), were also not affected by preincubation with either concentration of Ang II (Figure 1E). Of note, the endothelium-dependent vasodilator, acetylcholine, elicited concentration-dependent vasodilation, which was markedly reduced in ophthalmic arteries preincubated with Ang II 10^−7^ M, but not in those preincubated with Ang II 10^−9^ M and Ang II 10^−8^ M, respectively. The maximum increase in luminal diameter to acetylcholine was 85.20 ± 5.766%, 74.49 ± 8.006%, 59.95 ± 6.326%, and 91.68 ± 4.253% in arteries preincubated with Ang II 10^−9^ M, Ang II 10^−8^ M, Ang II 10^−7^ M or vehicle (* *p* < 0.05; Figure 1F). Of note, concentration–response curves to U46619, SNP, and acetylcholine in ophthalmic arteries preincubated overnight with vehicle solution were similar to those from freshly isolated arteries of mice that have been infused with vehicle solution for 7 days, suggesting that overnight incubation did not markedly affect vessel viability. Based on these results, Ang II 10^−7^ M was selected for induction of endothelial dysfunction in further experimental series.

### 3.2. Responses of Ophthalmic Arteries after AT1R and AT2R Blockade

**Figure 2:**
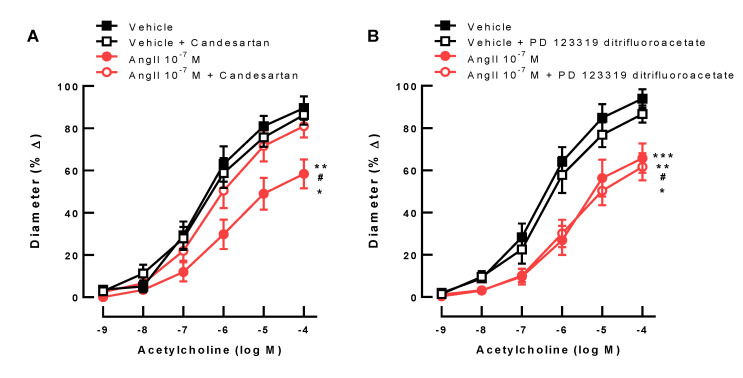
The AT1R blocker, candesartan, prevented Ang II-induced endothelial dysfunction (**A**) (*n* = 8 per group; ** *p* < 0.01, Ang II 10^−7^ M versus vehicle; # *p* < 0.01, Ang II 10^−7^ M versus vehicle + candesartan; * *p* < 0.05, Ang II 10^−7^ M versus Ang II 10^−7^ M + candesartan). The AT2R blocker, PD 123319 ditrifluoroacetate, had no effect on Ang II-induced endothelial dysfunction (**B**) (*n* = 8 per group; *** *p* < 0.001, Ang II 10^−7^ M + PD 123319 ditrifluoroacetate versus vehicle; ** *p* < 0.01, Ang II 10^−7^ M versus vehicle; # *p* < 0.01, vehicle + PD 123319 ditrifluoroacetate vs. Ang II 10^−7^ M + PD 123319 ditrifluoroacetate; * *p* < 0.05, vehicle + PD 123319 ditrifluoroacetate vs. Ang II 10^−7^ M). Data are expressed as mean ± SE.

Preincubation of ophthalmic arteries with vehicle plus the AT1R blocker, candesartan (10^−5^ M), for 22 h had no effect on responses to acetylcholine. However, preincubation with Ang II 10^−7^ M plus candesartan retained vasodilation responses to acetylcholine (Figure 2A). Preincubation of ophthalmic arteries with vehicle and the AT2R blocker, PD 123319 ditrifluoroacetate (10^−6^ M) did not affect cholinergic vasodilation (Figure 2B). Preincubation with Ang II 10^−7^ M and PD 123319 ditrifluoroacetate (10^−6^ M) did not prevent endothelial dysfunction in ophthalmic arteries.

### 3.3. Endothelial Function and ROS Formation

**Figure 3:**
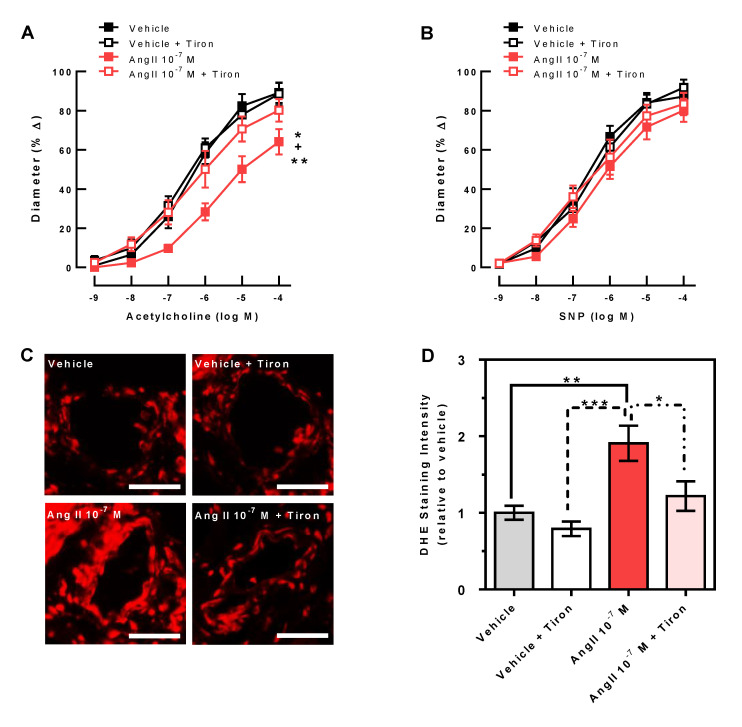
While Ang II 10^−7^ M reduced endothelium-dependent vasodilation (reduced acetylcholine responses), the ROS scavenger Tiron 10^−3^ M retained acetylcholine-induced vascular responses in Ang II 10^−7^ M-incubated ophthalmic arteries (**A**). Ang II and Tiron had no effect on the SNP response (endothelium-independent vasodilation) (**B**). Data are expressed as mean ± SE (*n* = 8 per group; ** *p* < 0.01, Ang II 10^−7^ M versus vehicle + Tiron; ^+^ *p* < 0.01, Ang II 10^−7^ M vs. Ang II 10^−7^ M + Tiron; * *p* < 0.05, Ang II 10^−7^ M versus vehicle). Ang II 10^−7^ M increased fluorescent intensity to DHE in ophthalmic artery cross-sections, indicative of elevated ROS levels, whereas Tiron prevented an Ang II-induced increase in DHE staining intensity (**C**,**D**). Data are expressed as mean ± SE (*n* = 8 per group; *** *p* < 0.001; ** *p* < 0.01; * *p* < 0.05). Scale bar = 50 µm.

While after incubation of ophthalmic arteries with Ang II 10^−7^ M responses to acetylcholine were markedly reduced, co-incubation of Ang II 10^−7^ M with the ROS scavenger, Tiron 10^−3^ M, did not impair acetylcholine-induced vascular reactivity (Figure 3A), indicating that endothelial function was retained when the ROS were quenched. Ang II and Tiron did not affect endothelium-independent vasodilatory responses to SNP (Figure 3B).

The staining of ophthalmic artery cross-sections with DHE revealed increased fluorescence intensity in arteries incubated with Ang II 10^−7^ M compared to cross-sections of arteries incubated with vehicle only, indicative of elevated ROS levels. However, co-incubation of Ang II 10^−7^ M with the ROS scavenger, Tiron 10^−3^ M, prevented the increase of DHE fluorescence intensity, suggesting that Tiron reduced oxidative stress in the vascular wall (Figure 3C,D, *n* = 8 per group).

### 3.4. Prooxidant Redox Enzyme Expression and Endothelial Function

**Figure 4:**
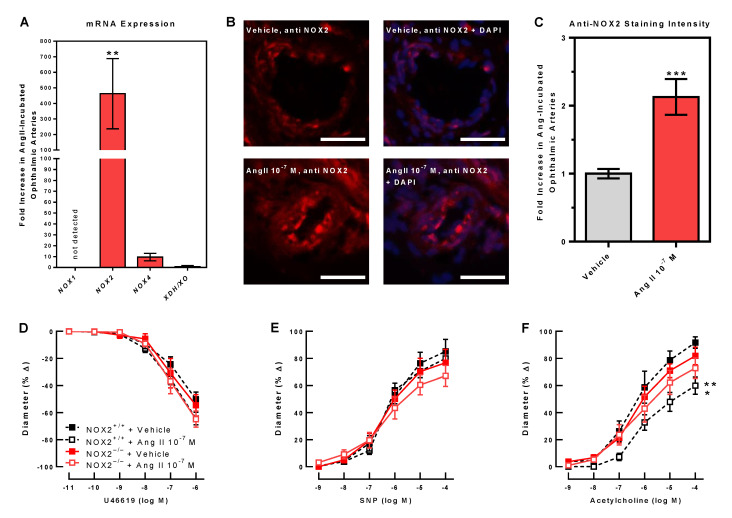
Incubation with Ang II (10^−7^ M) induced marked expression of *NOX2* mRNA in isolated ophthalmic arteries of C57Bl/6J mice. In contrast, *NOX1* mRNA was undetectable, and *NOX4* and *XDH*/*XO* mRNA only insignificantly increased in Ang II-incubated arteries compared to vehicle-incubated arteries (**A**) (*n* = 6 per group, ** *p* < 0.01 versus vehicle control). While immunoreactivity to NOX2 was only weak in vehicle-incubated ophthalmic arteries from C57Bl/6J mice (**B**,**C**), much stronger immunoreactivity to NOX2 was seen in the vascular wall of arteries incubated with Ang II 10^−7^ M (**B**,**C**) (*n* = 8 per group, *** *p* < 0.001, Ang 10^−7^ M versus vehicle control). Vasoconstrictor responses to U46619 (**D**), endothelium-independent vasodilation to SNP (**E**), and endothelium-dependent vasodilation to acetylcholine (**F**) were not reduced in isolated ophthalmic arteries from NOX2^–/–^ mice exposed to Ang II 10^−7^ M compared to vehicle, suggesting that Ang II failed to induce endothelial dysfunction when NOX2 was not present. The curves with dashed lines were reproduced from Figure 1D–F for the direct comparison with the respective control condition. Data are expressed as mean ± SE (*n* = 8 per group; ** *p* < 0.01, NOX2^+/+^ + Ang II 10^−7^ M versus NOX2^+/+^ + vehicle; * *p* < 0.05, NOX2^+/+^ + Ang II 10^−7^ M versus NOX2^–/–^ + vehicle). Scale bar = 50 µm.

In ophthalmic arteries incubated with Ang II 10^−7^ M, mRNA expression for the redox gene, *NOX2*, was markedly increased compared to vehicle-incubated arteries (Figure 4A). In contrast, *NOX1* mRNA was not detected in the ophthalmic arteries of either group. *NOX4* and *XO* mRNA expression were similar in ophthalmic arteries incubated with Ang II 10^−7^ M and vehicle. Immunoreactivity to the prooxidant redox enzyme, NOX2, has markedly increased in Ang II-incubated ophthalmic arteries (Figure 4B,C), suggesting that NOX2 expression was also induced on the protein level following Ang II 10^−7^ M incubation. To test whether NOX2 plays a functional role in mediating Ang II-induced endothelial dysfunction, ophthalmic arteries from NOX2^–/–^ mice were incubated with Ang II 10^−7^ M or vehicle. Notably, unlike in ophthalmic arteries from wild-type mice, responses to acetylcholine were retained in arteries from NOX2^–/–^ mice following Ang II 10^−7^ M incubation, suggesting that NOX2 is required for inducing endothelial dysfunction (Figure 4D–F).

### 3.5. Effects of Ang II on Vasodilatory Signaling Pathways

**Figure 5:**
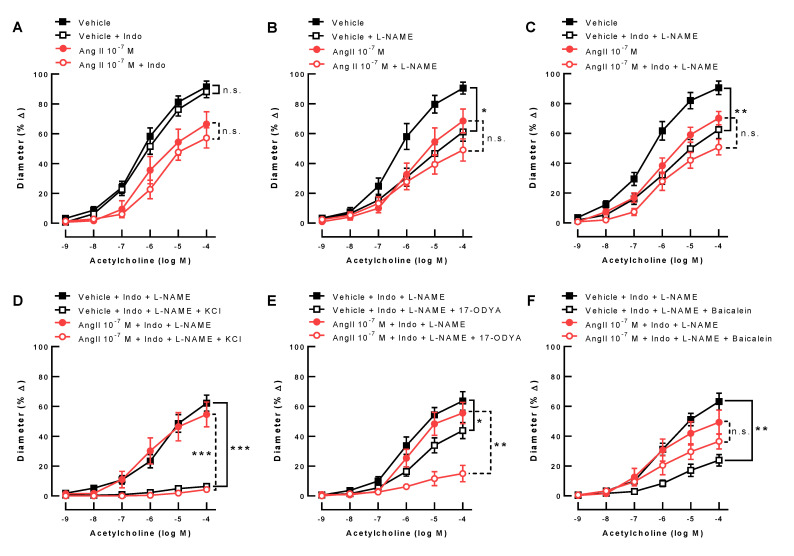
The non-isoform-selective COX blocker, indomethacin (Indo), had no effect on the acetylcholine-induced (endothelium-dependent) vasodilation in the vehicle-incubated and Ang II 10^−7^ M-incubated ophthalmic arteries from wild-type mice (C57BL/6J) (**A**). In contrast, the non-isoform-selective NOS inhibitor, L-NAME, markedly reduced the endothelium-dependent vasodilation in the vehicle-incubated arteries, but only had a negligible effect in Ang II-incubated arteries (**B**) (* *p* < 0.05, vehicle + L-NAME versus vehicle). Also, the combined blockade of COX and NOS significantly reduced endothelium-dependent vasodilation in vehicle-incubated but not in Ang II-incubated ophthalmic arteries (**C**) (** *p* < 0.01, vehicle + Indo + L-NAME versus vehicle). KCl (30 mM), which prevents hyperpolarization, virtually abolished endothelium-dependent vasodilation in both vehicle- and Ang II-incubated vessels, in which COX and NOS had been blocked, indicative of substantial EDHF involvement (**D**) (*** *p* < 0.001, vehicle + Indo + L-NAME + KCl versus vehicle + Indo + L-NAME and Ang II 10^−7^ M + Indo + L-NAME + KCl versus Ang II 10^−7^ M + Indo + L-NAME). Inhibition of CYP450 by 17-ODYA moderately blunted acetylcholine-induced vasodilation in vehicle-incubated ophthalmic arteries. Remarkably, the blocking effect was much more pronounced in the Ang II-exposed arteries (**E**) (* *p* < 0.05, vehicle + Indo + L-NAME + 17-ODYA versus vehicle + Indo + L-NAME; ** *p* < 0.01, Ang II 10^−7^ M + Indo + L-NAME + 17-ODYA versus Ang II 10^−7^ M + Indo + L-NAME). The 12/15-LOX inhibitor, baicalein, markedly reduced endothelium-dependent vasodilation in the vehicle-treated ophthalmic arteries, while having no significant effect on Ang II-treated ophthalmic arteries (**F**) (** *p* < 0.01, vehicle + Indo + L-NAME + Baicalein versus vehicle + Indo + L-NAME). Data are expressed as mean ± SE (*n* = 8 per group).

Incubation with the non-isoform-selective COX blocker, indomethacin, did not affect acetylcholine-induced vasodilation either in ophthalmic arteries incubated with vehicle or with Ang II 10^−7^ M (Figure 5A). In contrast, the non-isoform-selective NOS inhibitor, L-NAME, markedly reduced responses to acetylcholine in vehicle-incubated but not in Ang II-incubated ophthalmic arteries, suggesting that NOS involvement was smaller in Ang II-treated ophthalmic arteries (Figure 5B). The combination of indomethacin and L-NAME reduced acetylcholine-induced to a similar extent as L-NAME alone in vehicle-treated arteries but had no significant effect in Ang II-treated ophthalmic arteries (Figure 5C), suggesting that COX and NOS did not compensate for each other in ophthalmic arteries, as opposed to previous observations in retinal arterioles [47].

The portion of the vasodilatory response that remained after combined incubation with indomethacin and L-NAME was virtually abolished by the addition of 30 mM KCl, indicative of EDHF involvement in both vehicle- and Ang II-incubated ophthalmic artery segments (Figure 5D). Interestingly, blockade of CYP450 pathways with 17-ODYA only moderately reduced vasodilatory responses in ophthalmic arteries from vehicle-incubated arteries but largely in Ang II-incubated arteries (Figure 5E), suggesting a larger contribution of CYP450 metabolites following Ang II exposure. In contrast, the 12/15-LOX inhibitor, baicalein, markedly reduced responses to acetylcholine in vehicle-incubated ophthalmic arteries but had no significant effect in Ang II-treated ophthalmic arteries (Figure 5F), indicative of a negligible contribution of LOX metabolites to endothelium-dependent vasodilation following Ang II exposure.

## 4. Discussion

There are several major new findings emerging from the present study. First, in vivo exposure of ophthalmic arteries to Ang II resulted in impaired vasodilatory responses to acetylcholine, but not to SNP, indicative of endothelial dysfunction. Likewise, Ang II concentration-dependently induced endothelial dysfunction in vitro. This effect was prevented by the AT1R blocker, candesartan, but not by the AT2R blocker, PD 123319 ditrifluoroacetate, suggesting that Ang II-induced endothelial dysfunction was mediated via the AT1R. Second, incubation of isolated ophthalmic arteries with Ang II resulted in elevated ROS levels in the vascular wall, which was prevented by coincubation with the ROS scavenger, Tiron, that also prevented Ang II-induced endothelial dysfunction, indicative of ROS involvement. Third, Ang II exposure of ophthalmic arteries from C57BL/6J mice elevated expression of the prooxidant enzyme, NOX2, in the vascular wall both at the mRNA and protein level. Remarkably, in arteries from NOX2^–/–^ mice, Ang II failed to induce endothelial dysfunction confirming that NOX2-mediated ROS formation is involved in Ang II-induced endothelial dysfunction in the ophthalmic artery. Fourth, Ang II exposure reduced the contribution of NOS and LOX metabolites but enhanced the contribution of CYP450 products to endothelium-dependent vasodilation, suggesting that the individual endothelial signaling pathways have different susceptibilities to Ang II exposure.

Previous studies in non-ocular vascular beds of various disease models, such as arterial hypertension and diabetes, revealed that Ang II triggers vascular endothelial dysfunction by inducing oxidative stress via involvement of the AT1R [2,48,49]. The present study is the first to determine the mechanisms of Ang II-induced endothelial dysfunction in ocular blood vessels. We found that activation of the AT1R is required to induce endothelial dysfunction in the ophthalmic artery. Other studies in ocular vascular cells and tissues reported that activation of the AT1R by Ang II-mediated a variety of pathophysiological actions, such as proliferation and migration of ciliary artery smooth muscle cells and VEGF-induced cell sprouting and migration in retinal vascular endothelial cells [19,20,21]. In the diabetic retina, AT1R activation by angiotensin II was shown to induce NFκB-dependent proinflammatory signaling pathways and leukocyte adhesion to blood vessels, thus, contributing to retinal inflammation [50,51]. Moreover, in a rat model of retinal ischemia, AT1R-mediated signaling was shown to contribute to retinal microglia activation [52].

In various non-ocular vascular beds, Ang II was shown to induce oxidative stress and endothelial dysfunction via involvement of the prooxidant redox enzyme, NOX2, mitochondrial enzymes, and uncoupled endothelial nitric oxide synthase (eNOS) [53,54,55,56]. The present study provides evidence that NOX2 is critical for Ang II-induced endothelial dysfunction in ophthalmic arteries. We previously reported that NOX2 contributes to endothelial dysfunction in a variety of ocular disease models, such as acute respiratory distress syndrome, ischemia-reperfusion injury, hypercholesterolemia, and glaucoma, highlighting the critical pathophysiological role of NOX2 in the ocular vascular system [44,46,57,58].

Furthermore, the present study is the first to give insight into the vasodilatory endothelial signaling pathways affected by Ang II in ocular blood vessels. In vehicle-exposed ophthalmic arteries, endothelium-dependent vasodilation was driven by NOS and EDHFs. The EDHF portion was mediated by CYP450 and 12/15-LOX metabolites. These findings are in agreement with previous observations in freshly isolated C57BL/6J mouse ophthalmic arteries, suggesting that overnight incubation of vessels had no marked effect on the contribution of endothelial signaling pathways to vasodilation [41].

In contrast, the contribution of NOS to endothelium-dependent vasodilation was negligible following Ang II incubation, suggesting that NOS was dysfunctional in these arteries. In support of this concept, previous studies in cultured vascular endothelial cells, isolated blood vessels, and experimental animals revealed that Ang II exposure induces eNOS uncoupling to produce superoxide instead of nitric oxide [53,59,60]. Of note, in Ang II-exposed ophthalmic arteries, endothelium-dependent vasodilation was mediated predominantly by EDHFs. However, in contrast to ophthalmic arteries incubated with vehicle, EDHF-driven vasodilation was mediated predominantly by CYP450 metabolites in Ang II-exposed arteries, suggesting that the contribution of the 12/15-LOX pathway became negligible. In support of this concept, previous studies reported that some AT1R blockers, such as telmisartan, exerted inhibitory effects on CYP450 enzymes involved in the generation of epoxyeicosatrienoic acids (EETs), which play an important role in mediating EDHF-dependent vasodilation, suggesting that, conversely, CYP450 pathways may be activated by Ang II [61,62].

Based on these findings, CYP450-dependent signaling pathways appear to be of major importance for endothelium-dependent vasodilation of ophthalmic arteries following Ang II exposure whereas NOS and 12/15-LOX signaling become impaired.

**Figure 6:**
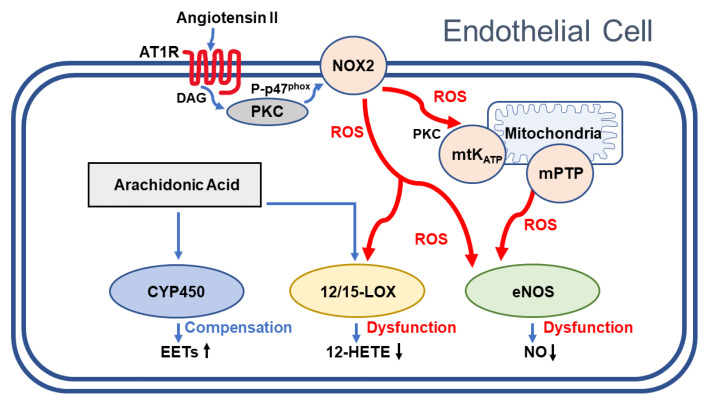
Proposed hypothetical signaling pathways affected by Ang II in the mouse ophthalmic artery endothelium. Activation of the angiotensin receptor type 1 (AT1R) by angiotensin II activates NADPH oxidase 2 (NOX2) in the cytoplasmic membrane via diacylglycerol (DAG), protein kinase C (PKC), and phosphorylation of the NADPH oxidase subunit p47^phox^ to P-p47^phox^. Moreover, NOX2-derived ROS were shown to open mitochondrial KATP channels (mtKATP) in the mitochondrial membrane subsequently causing the opening of permeability transition pores (mPTP) providing an amplification mechanism for Ang II-induced oxidative stress. The ROS signaling mechanism is summarized from previous publications [9,56,64]. Based on our findings, NOX2-derived reactive oxygen species (ROS) inactivate endothelial nitric oxide synthase (eNOS), which leads to diminished nitric oxide (NO) production. Moreover, impairment of 12/15-lipoxygenase (12/15-LOX) by ROS may blunt the generation of 12-hydroxyeicosatetraenoic acid (12-HETE), which is the predominant HETE regioisomer in the mouse and which appears to act as an endothelium-derived hyperpolarizing factor (EDHF) [65]. In contrast, cytochrome P450 oxygenases (CYP450), which contribute to EDHF-mediated vasodilation by the generation of epoxyeicosatrienoic acids (EETs), appears to be unaffected by Ang II-induced ROS formation and appears to compensate for the dysfunctional eNOS- and 12/15 LOX-dependent signaling pathways.

In a previous study that used isoform-selective NOS inhibitors in mutant mice lacking individual NOS isoforms, we observed that eNOS is the only NOS isoform contributing to acetylcholine-induced vasodilation in ophthalmic arteries [63]. Interestingly, in ophthalmic arteries from mice devoid of eNOS, the contribution of 12/15-LOX metabolites to endothelium-dependent vasodilation even increased while the influence of CYP450 products decreased, suggesting that the reduced NO production by eNOS is not the driving force for the compensation by CYP450 products in Ang II-exposed ophthalmic arteries [42]. Based on our findings, we propose the following scheme demonstrating how endothelial signaling mechanisms might be affected by Ang II in the mouse ophthalmic artery (Figure 6).

The present study shows that the endothelial vasodilatory signaling pathways are largely preserved in ophthalmic arteries after 22 h of in vitro incubation with vehicle only, because the signaling mechanisms did not differ from those observed previously in freshly isolated arteries, suggesting that this in vitro model may be useful to test also other signaling pathways in ophthalmic arteries [41]. However, it has to be noted that the mechanisms of angiotensin II-induced vascular dysfunction may be more complex in vivo. It has previously been shown that in vivo recruitment of immune cells, particularly monocytes and macrophages, into the vascular wall substantially contributes to angiotensin II-induced vascular endothelial dysfunction, inflammation, and hypertension [6,53,66]. Moreover, angiotensin II-induced vascular dysfunction and arterial hypertension were shown to be modulated by gut microbiota, emphasizing the complexity of the involved mechanisms [67].

Notably, our ophthalmic artery preparation model demonstrates that angiotensin II induces endothelial dysfunction independently of blood pressure changes and largely independent on immune cells, since only resident immune cells from the vascular wall and adventitial tissue may have been recruited by Ang II activation to promote inflammation in this in vitro model.

From a clinical point of view, blockers of angiotensin-converting enzyme and AT1R may exert beneficial effects in ocular diseases associated with endothelial dysfunction. For example, in diabetic retinopathy, Ang II, oxidative stress, and inflammation was shown to play an important pathophysiological role [68]. Likewise, alterations of the RAS have been implicated in the pathophysiology of age-related macular degeneration and glaucoma, which both have also been associated with vascular endothelial dysfunction [31,34,46,69,70,71]. Hence, the role of the RAS deserves to be pursued further in these diseases.

## 5. Conclusions

In conclusion, both in vivo and in vitro exposure to Ang II induces endothelial dysfunction in mouse ophthalmic arteries. The effect is mediated primarily by the AT1R, NOX2, and ROS. In addition, eNOS-dependent and LOX-dependent vasodilation is impaired while the contribution of CYP450 metabolites is enhanced after Ang II exposure. From a clinical point of view, blockade of Ang II production or AT1R signaling and/or NOX2 may be helpful to retain or restore vascular endothelial function in various age-related diseases associated with endothelial dysfunction in ocular blood vessels, such as age-related macular degeneration, diabetic retinopathy, and glaucoma. Since CYP450-dependent signaling pathways appear to compensate for dysfunctional NOS and 12/15-LOX during chronic exposure to high Ang II levels, additional blockade of CYP450 may further reduce endothelial function under such pathophysiological conditions.

## Figures and Tables

**Table 1:** Primer sequences used for quantitative PCR analysis in ophthalmic arteries.

Gene	Accession Number	Forward	Reverse
*NOX1*	NM_172203.2	GGTTGGGGCTGAACATTTTTC	TCGACACACAGGAATCAGGAT
*NOX2*	NM_007807.5	GCACCTGCAGCCTGCCTGAATT	TTGTGTGGATGGCGGTGTGCA
*NOX4*	NM_015760.5	GGCTGGCCAACGAAGGGGTTAA	GAGGCTGCAGTTGAGGTTCAGGACA
*XDH*	NM_011723.3	CGATGACGAGGACAACGGTA	TGAAGGCGGTCATACTTGGAG
*ACTB*	NM_007393.5	CACCCGCGAGCACAGCTTCTTT	AATACAGCCCGGGGAGCATC

## Data Availability

Data is contained within the article.

## References

[1] Colafella K.M.M., Bovée D.M., Danser A.J. (2019). The renin-angiotensin-aldosterone system and its therapeutic targets. Exp. Eye Res..

[2] Rajagopalan S., Kurz S., Münzel T., Tarpey M., Freeman B.A., Griendling K., Harrison D.G. (1996). Angiotensin II-mediated hypertension in the rat increases vascular superoxide production via membrane NADH/NADPH oxidase activation. Contribution to alterations of vasomotor tone. J. Clin. Investig..

[3] Wang D., Chen Y., Chabrashvili T., Aslam S., Conde L.J.B., Umans J.G., Wilcox C.S. (2003). Role of Oxidative Stress in Endothelial Dysfunction and Enhanced Responses to Angiotensin II of Afferent Arterioles from Rabbits Infused with Angiotensin II. J. Am. Soc. Nephrol..

[4] Gomolak J.R., Didion S.P. (2014). Angiotensin II-induced endothelial dysfunction is temporally linked with increases in interleukin-6 and vascular macrophage accumulation. Front. Physiol..

[5] Zhang Z., Wang M., Xue S.J., Liu D.H., Tang Y.B. (2012). Simvastatin ameliorates angiotensin II-induced endothelial dysfunction through restoration of Rho-BH4-eNOS-NO pathway. Cardiovasc. Drugs Ther..

[6] Wenzel P., Knorr M., Kossmann S., Stratmann J., Hausding M., Schuhmacher S., Karbach S.H., Schwenk M., Yogev N., Schulz E. (2011). Lysozyme M-positive monocytes mediate angiotensin II-induced arterial hypertension and vascular dysfunction. Circulation.

[7] Oelze M., Kröller-Schön S., Steven S., Lubos E., Doppler C.E.J., Hausding M., Tobias S., Brochhausen C., Li H., Torzewski M. (2014). Glutathione Peroxidase-1 Deficiency Potentiates Dysregulatory Modifications of Endothelial Nitric Oxide Synthase and Vascular Dysfunction in Aging. Hypertension.

[8] Wenzel P., Schuhmacher S., Kienhöfer J., Müller J., Hortmann M., Oelze M., Schulz E., Treiber N., Kawamoto T., Scharffetter-Kochanek K. (2008). Manganese superoxide dismutase and aldehyde dehydrogenase deficiency increase mitochondrial oxidative stress and aggravate age-dependent vascular dysfunction. Cardiovasc. Res..

[9] Kröller-Schön S., Steven S., Kossmann S., Scholz A., Daub S., Oelze M., Xia N., Hausding M., Mikhed Y., Zinßius E. (2014). Molecular Mechanisms of the Crosstalk Between Mitochondria and NADPH Oxidase Through Reactive Oxygen Species—Studies in White Blood Cells and in Animal Models. Antioxid. Redox Signal..

[10] Senanayake P.D., Drazba J., Shadrach K., Milsted A., Rungger-Brandle E., Nishiyama K., Miura S.-I., Karnik S., Sears J.E., Hollyfield J.G. (2007). Angiotensin II and Its Receptor Subtypes in the Human Retina. Investig. Opthalmology Vis. Sci..

[11] Ferrari-DiLeo G., Davis E.B., Anderson D.R. (1991). Angiotensin II binding receptors in retinal and optic nerve head blood vessels. An autoradiographic approach. Investig. Ophthalmol. Vis. Sci..

[12] Sato T., Niwa M., Himeno A., Tsutsumi K., Amemiya T. (1993). Quantitative receptor autoradiographic analysis for angiotensin II receptors in bovine retinal microvessels: Quantitation with radioluminography. Cell Mol. Neurobiol..

[13] Rockwood E.J., Fantes F., Davis E.B., Anderson D.R. (1987). The response of retinal vasculature to angiotensin. Investig. Ophthalmol. Vis. Sci..

[14] Nyborg N.C., Nielsen P.J. (1990). Angiotensin-II contracts isolated human posterior ciliary arteries. Investig. Ophthalmol. Vis. Sci..

[15] Nyborg N.C., Nielsen P.J., Prieto D., Benedito S. (1990). Angiotensin II does not contract bovine retinal resistance arteries in vitro. Exp. Eye Res..

[16] Meyer P., Flammer J., Lüscher T.F. (1995). Local action of the renin angiotensin system in the porcine ophthalmic circulation: Effects of ACE-inhibitors and angiotensin receptor antagonists. Investig. Ophthalmol. Vis. Sci..

[17] Wakisaka M., Yoshinari M., Nakamura S., Asano T., Sonoki K., Shi A.-H., Iwase M., Takata Y., Fujishima M. (1999). Suppression of Sodium-Dependent Glucose Uptake by Captopril Improves High-Glucose-Induced Morphological and Functional Changes of Cultured Bovine Retinal Pericytes. Microvasc. Res..

[18] Nadal J., Scicli G., Carbini L., Nussbaum J., Scicli A. (1999). Angiotensin II and Retinal Pericytes Migration. Biochem. Biophys. Res. Commun..

[19] Dubey R.K., Flammer J., Lüscher T.F. (1998). Angiotensin II and insulin induce growth of ciliary artery smooth muscle: Effects of AT1/AT2 antagonists. Investig. Ophthalmol. Vis. Sci..

[20] Carbajo-Lozoya J., Lutz S., Feng Y., Kroll J., Hammes H.-P., Wieland T. (2012). Angiotensin II modulates VEGF-driven angiogenesis by opposing effects of type 1 and type 2 receptor stimulation in the microvascular endothelium. Cell. Signal..

[21] Otani A., Takagi H., Suzuma K., Honda Y. (1998). Angiotensin II potentiates vascular endothelial growth factor-induced angiogenic activity in retinal microcapillary endothelial cells. Circ. Res..

[22] Otani A., Takagi H., Oh H., Suzuma K., Matsumura M., Ikeda E., Honda Y. (2000). Angiotensin II-stimulated vascular endothelial growth factor expression in bovine retinal pericytes. Investig. Ophthalmol. Vis. Sci..

[23] Larsen M., Hommel E., Parving H.-H., Lund-Andersen H. (1990). Protective effect of captopril on the blood-retina barrier in normotensive insulin-dependent diabetic patients with nephropathy and background retinopathy. Graefe’s Arch. Clin. Exp. Ophthalmol..

[24] Moravski C.J., Kelly D., Cooper M.E., Gilbert R.E., Bertram J.F., Shahinfar S., Skinner S.L., Wilkinson-Berka J.L. (2000). Retinal neovascularization is prevented by blockade of the renin-angiotensin system. Hypertension.

[25] Kim J.H., Kim J.H., Yu Y.S., Cho C.S., Kim K.-W. (2008). Blockade of Angiotensin II Attenuates VEGF-Mediated Blood—Retinal Barrier Breakdown in Diabetic Retinopathy. Br. J. Pharmacol..

[26] Nakamura H., Yamazaki M., Ohyama T., Inoue T., Arakawa N., Domon Y., Yokoyama T. (2009). Role of Angiotensin II Type 1 Receptor on Retinal Vascular Leakage in a Rat Oxygen-Induced Retinopathy Model. Ophthalmic Res..

[27] Nath M., Chandra P., Halder N., Singh B., Deorari A.K., Kumar A., Azad R., Velpandian T. (2016). Involvement of Renin-Angiotensin System in Retinopathy of Prematurity—A Possible Target for Therapeutic Intervention. PLoS ONE.

[28] Okunuki Y., Usui Y., Nagai N., Kezuka T., Ishida S., Takeuchi M., Goto H. (2009). Suppression of Experimental Autoimmune Uveitis by Angiotensin II Type 1 Receptor Blocker Telmisartan. Investig. Opthalmology Vis. Sci..

[29] Qiu Y., Tao L., Zheng S., Lin R., Fu X., Chen Z., Lei C., Wang J., Li H., Li Q. (2016). AAV8-Mediated Angiotensin-Converting Enzyme 2 Gene Delivery Prevents Experimental Autoimmune Uveitis by Regulating MAPK, NF-κB and STAT3 Pathways. Sci. Rep..

[30] Fukuda K., Hirooka K., Mizote M., Nakamura T., Itano T., Shiraga F., Tung C., Aquavella J., Zavislan J., Koh S. (2010). Neuroprotection against Retinal Ischemia–Reperfusion Injury by Blocking the Angiotensin II Type 1 Receptor. Investig. Opthalmology Vis. Sci..

[31] Semba K., Namekata K., Guo X., Harada C., Harada T., Mitamura Y. (2014). Renin–angiotensin system regulates neurodegeneration in a mouse model of normal tension glaucoma. Cell Death Dis..

[32] Quigley H.A., Pitha I.F., Welsbie D.S., Nguyen C., Steinhart M., Nguyen T.D., Pease M., Oglesby E.N., Berlinicke C.A., Mitchell K.L. (2015). Losartan Treatment Protects Retinal Ganglion Cells and Alters Scleral Remodeling in Experimental Glaucoma. PLoS ONE.

[33] Hazlewood R.J., Chen Q., Clark F.K., Kuchtey J., Kuchtey R.W. (2018). Differential effects of angiotensin II type I receptor blockers on reducing intraocular pressure and TGFbeta signaling in the mouse retina. PLoS ONE.

[34] Nagai N., Kawashima H., Toda E., Homma K., Osada H., Guzman N.A., Shibata S., Uchiyama Y., Okano H., Tsubota K. (2020). Renin–angiotensin system impairs macrophage lipid metabolism to promote age-related macular degeneration in mouse models. Commun. Biol..

[35] Didion S.P., Kinzenbaw D.A., Faraci F.M. (2005). Critical role for CuZn-superoxide dismutase in preventing angiotensin II-induced endothelial dysfunction. Hypertension.

[36] Schrader L.I., Kinzenbaw D.A., Johnson A.W., Faraci F.M., Didion S.P. (2007). IL-6 Deficiency Protects Against Angiotensin II–Induced Endothelial Dysfunction and Hypertrophy. Arter. Thromb. Vasc. Biol..

[37] Helmstädter J., Frenis K., Filippou K., Grill A., Dib M., Kalinovic S., Pawelke F., Kus K., Kröller-Schön S., Oelze M. (2020). Endothelial GLP-1 (Glucagon-Like Peptide-1) Receptor Mediates Cardiovascular Protection by Liraglutide In Mice With Experimental Arterial Hypertension. Arter. Thromb. Vasc. Biol..

[38] Pollock J.D., Williams D.A., Gifford M.A., Li L.L., Du X., Fisherman J., Orkin S.H., Doerschuk C.M., Dinauer M.C. (1995). Mouse model of X–linked chronic granulomatous disease, an inherited defect in phagocyte superoxide production. Nat. Genet..

[39] Gericke A., Mayer V.G.A., Steege A., Patzak A., Neumann U., Grus F.H., Joachim S.C., Choritz L., Wess J., Pfeiffer N. (2009). Cholinergic responses of ophthalmic arteries in M3 and M5 muscarinic acetylcholine receptor knockout mice. Investig. Opthalmology Vis. Sci..

[40] Gericke A., Steege A., Manicam C., Böhmer T., Wess J., Pfeiffer N. (2014). Role of the M3 Muscarinic Acetylcholine Receptor Subtype in Murine Ophthalmic Arteries After Endothelial Removal. Investig. Opthalmology Vis. Sci..

[41] Manicam C., Staubitz J., Brochhausen C., Grus F.H., Pfeiffer N., Gericke A. (2016). The Gatekeepers in the Mouse Ophthalmic Artery: Endothelium-Dependent Mechanisms of Cholinergic Vasodilation. Sci. Rep..

[42] Manicam C., Ginter N., Li H., Xia N., Goloborodko E., Zadeh J.K., Musayeva A., Pfeiffer N., Gericke A. (2017). Compensatory Vasodilator Mechanisms in the Ophthalmic Artery of Endothelial Nitric Oxide Synthase Gene Knockout Mice. Sci. Rep..

[43] Frenis K., Helmstädter J., Ruan Y., Schramm E., Kalinovic S., Kröller-Schön S., Jimenez M.T.B., Hahad O., Oelze M., Jiang S. (2021). Ablation of lysozyme M-positive cells prevents aircraft noise-induced vascular damage without improving cerebral side effects. Basic Res. Cardiol..

[44] Zadeh J.K., Ruemmler R., Hartmann E.K., Ziebart A., Ludwig M., Patzak A., Xia N., Li H., Pfeiffer N., Gericke A. (2019). Responses of retinal arterioles and ciliary arteries in pigs with acute respiratory distress syndrome (ARDS). Exp. Eye Res..

[45] Schröder K., Vecchione C., Jung O., Schreiber J.G., Shiri-Sverdlov R., van Gorp P.J., Busse R., Brandes R.P. (2006). Xanthine oxidase inhibitor tungsten prevents the development of atherosclerosis in ApoE knockout mice fed a Western-type diet. Free. Radic. Biol. Med..

[46] Gericke A., Mann C., Zadeh J.K., Musayeva A., Wolff I., Wang M., Pfeiffer N., Daiber A., Li H., Xia N. (2019). Elevated Intraocular Pressure Causes Abnormal Reactivity of Mouse Retinal Arterioles. Oxidative Med. Cell. Longev..

[47] Gericke A., Wolff I., Musayeva A., Zadeh J.K., Manicam C., Pfeiffer N., Li H., Xia N. (2019). Retinal arteriole reactivity in mice lacking the endothelial nitric oxide synthase (eNOS) gene. Exp. Eye Res..

[48] Welch W.J., Wilcox C.S. (2001). AT1 receptor antagonist combats oxidative stress and restores nitric oxide signaling in the SHR. Kidney Int..

[49] Wong W.T., Tian X.Y., Xu A., Ng C.F., Lee H.K., Chen Z.Y., Au C.L., Yao X., Huang Y. (2010). Angiotensin II Type 1 Receptor-Dependent Oxidative Stress Mediates Endothelial Dysfunction in Type 2 Diabetic Mice. Antioxid. Redox Signal..

[50] Nagai N., Izumi-Nagai K., Oike Y., Koto T., Satofuka S., Ozawa Y., Yamashiro K., Inoue M., Tsubota K., Umezawa K. (2007). Suppression of diabetes-induced retinal inflammation by blocking the angiotensin II type 1 receptor or its downstream nuclear factor-kappaB pathway. Investig. Ophthalmol. Vis. Sci..

[51] Chen P., Guo A.M., Edwards P.A., Trick G., Scicli A.G. (2007). Role of NADPH oxidase and ANG II in diabetes-induced retinal leukostasis. Am. J. Physiol. Integr. Comp. Physiol..

[52] Rana I., Suphapimol V., Jerome J.R., Talia D.M., Deliyanti D., Wilkinson-Berka J.L. (2020). Angiotensin II and aldosterone activate retinal microglia. Exp. Eye Res..

[53] Kossmann S., Hu H., Steven S., Schönfelder T., Fraccarollo D., Mikhed Y., Brähler M., Knorr M., Brandt M., Karbach S.H. (2014). Inflammatory Monocytes Determine Endothelial Nitric-oxide Synthase Uncoupling and Nitro-oxidative Stress Induced by Angiotensin II. J. Biol. Chem..

[54] Kroller-Schon S., Jansen T., Schuler A., Oelze M., Wenzel P., Hausding M., Kerahrodi J.G., Beisele M., Lackner K.J., Daiber A. (2013). Peroxisome proliferator-activated receptor gamma, coactivator 1alpha deletion induces angiotensin II-associated vascular dysfunction by increasing mitochondrial oxidative stress and vascular inflammation. Arterioscler. Thromb. Vasc. Biol..

[55] Chrissobolis S., Banfi B., Sobey C.G., Faraci F.M. (2012). Role of Nox isoforms in angiotensin II-induced oxidative stress and endothelial dysfunction in brain. J. Appl. Physiol..

[56] Dikalov S.I., Nazarewicz R.R., Bikineyeva A., Hilenski L., Lassègue B., Griendling K., Harrison D.G., Dikalova A.E. (2014). Nox2-Induced Production of Mitochondrial Superoxide in Angiotensin II-Mediated Endothelial Oxidative Stress and Hypertension. Antioxid. Redox Signal..

[57] Zadeh J.K., Garcia-Bardon A., Hartmann E.K., Pfeiffer N., Omran W., Ludwig M., Patzak A., Xia N., Li H., Gericke A. (2019). Short-Time Ocular Ischemia Induces Vascular Endothelial Dysfunction and Ganglion Cell Loss in the Pig Retina. Int. J. Mol. Sci..

[58] Zadeh J.K., Zhutdieva M.B., Laspas P., Yüksel C., Musayeva A., Pfeiffer N., Brochhausen C., Oelze M., Daiber A., Xia N. (2019). Apolipoprotein E Deficiency Causes Endothelial Dysfunction in the Mouse Retina. Oxidative Med. Cell. Longev..

[59] Galougahi K.K., Liu C.C., Gentile C., Kok C., Nunez A., Garcia A., Fry N.A., Davies M.J., Hawkins C.L., Rasmussen H.H. (2014). Glutathionylation mediates angiotensin II-induced eNOS uncoupling, amplifying NADPH oxidase-dependent endothelial dysfunction. J. Am. Heart Assoc..

[60] Oak J.-H., Cai H. (2006). Attenuation of Angiotensin II Signaling Recouples eNOS and Inhibits Nonendothelial NOX Activity in Diabetic Mice. Diabetes.

[61] Senda A., Mukai Y., Hayakawa T., Kato Y., Eliasson E., Rane A., Toda T., Inotsume N. (2017). Angiotensin II Receptor Blockers Inhibit the Generation of Epoxyeicosatrienoic Acid from Arachidonic Acid in Recombinant CYP2C9, CYP2J2 and Human Liver Microsomes. Basic Clin. Pharmacol. Toxicol..

[62] Kato Y., Mukai Y., Rane A., Inotsume N., Toda T. (2017). The Inhibitory Effect of Telmisartan on the Metabolism of Arachidonic Acid by CYP2C9 and CYP2C8: An in Vitro Study. Biol. Pharm. Bull..

[63] Laspas P., Goloborodko E., Sniatecki J.J., Kordasz M.L., Manicam C., Wojnowski L., Li H., Patzak A., Pfeiffer N., Gericke A. (2014). Role of nitric oxide synthase isoforms for ophthalmic artery reactivity in mice. Exp. Eye Res..

[64] Schulz E., Wenzel P., Münzel T., Daiber A. (2014). Mitochondrial Redox Signaling: Interaction of Mitochondrial Reactive Oxygen Species with Other Sources of Oxidative Stress. Antioxid. Redox Signal..

[65] Gauthier K.M., Goldman D.H., Aggarwal N.T., Chawengsub Y., Falck J., Campbell W.B. (2011). Role of arachidonic acid lipoxygenase metabolites in acetylcholine-induced relaxations of mouse arteries. Am. J. Physiol. Circ. Physiol..

[66] De Ciuceis C., Amiri F., Brassard P., Endemann D.H., Touyz R.M., Schiffrin E.L. (2005). Reduced vascular remodeling, endothelial dysfunction, and oxidative stress in resistance arteries of angiotensin II-infused macrophage colony-stimulating factor-deficient mice: Evidence for a role in inflammation in angiotensin-induced vascular injury. Arterioscler. Thromb. Vasc. Biol..

[67] Karbach S.H., Schönfelder T., Brandão I., Wilms E., Hörmann N., Jäckel S., Schüler R., Finger S., Knorr M., Lagrange J. (2016). Gut Microbiota Promote Angiotensin II–Induced Arterial Hypertension and Vascular Dysfunction. J. Am. Hear. Assoc..

[68] Wang M.-H., Hsiao G., Al-Shabrawey M. (2020). Eicosanoids and Oxidative Stress in Diabetic Retinopathy. Antioxidants.

[69] Holappa M., Vapaatalo H., Vaajanen A. (2020). Local ocular renin–angiotensin–aldosterone system: Any connection with intraocular pressure? A comprehensive review. Ann. Med..

[70] Resch H., Garhöfer G., Hommer A., Schmetterer L., Fuchsjäger-Mayrl G. (2009). Endothelial dysfunction in glaucoma. Acta Ophthalmol..

[71] Ruan Y., Jiang S., Gericke A. (2021). Age-Related Macular Degeneration: Role of Oxidative Stress and Blood Vessels. Int. J. Mol. Sci..

